# Direct epitaxy of wafer-scale 2D vdW heterostructures

**DOI:** 10.1093/nsr/nwaf209

**Published:** 2025-05-22

**Authors:** Lain-Jong Li

**Affiliations:** Department of Materials Science and Engineering, National University of Singapore, Singapore

Two-dimensional (2D) van der Waals (vdW) heterostructures, formed by stacking different 2D materials, have become promising candidates for precise control over electronic properties due to their adjustable band structures. For instance, modifying the composition and stacking order of the 2D materials enables a wide range of bandgap tuning, which supports various applications in electronic and optoelectronic devices. Moreover, their atomically sharp interfaces promote efficient charge transfer, minimizing carrier scattering and improving device performance [1,2].

However, the large-scale production of high-quality 2D vdW heterostructures has proven to be quite challenging. Current methods, such as mechanical transfer and sulfurization/selenization, face significant obstacles. Although mechanical transfer can yield high-purity isolated flakes, it is plagued by issues like interfacial impurities, wrinkles, and cracks resulting from the transfer process. These defects can disrupt or decouple the electronic properties of the heterostructures. On the other hand, while sulfurization and selenization have the potential for low-temperature formation [3], they often encounter challenges like insufficient crystallinity and lack of control over material orientation. Consequently, the resulting heterostructures frequently display non-uniform properties, which can adversely affect their performance [4,5].

To tackle these persistent challenges, a recent work published in *National Science Review* [6] introduces a significant advancement by employing direct epitaxial growth for wafer-scale SnS_2_/MoS_2_ 2D vdW heterostructures. This method effectively addresses the crucial issue of achieving both high quality and large-scale production. Traditionally, the growth of such heterostructures has struggled with ensuring uniform nucleation and growth. A notable innovation in this approach is the enhancement of adsorption interactions between the intermediates and the substrate.

Specifically, the introduction of hydrogen plays a vital role in breaking Sn-O bonds in SnO_2_. Acting as a reducing agent, hydrogen facilitates the formation of smaller SnO_2_H_2_ clusters, which are more prone to being absorbed on the MoS_2_ surface. This leads to a uniform distribution of Sn precursors and, consequently, uniform nucleation of the SnS_2_. In contrast, in the absence of hydrogen, the growth intermediates tend to aggregate, resulting in uneven and often defective structures (Fig. 1a).

**Figure 1.**
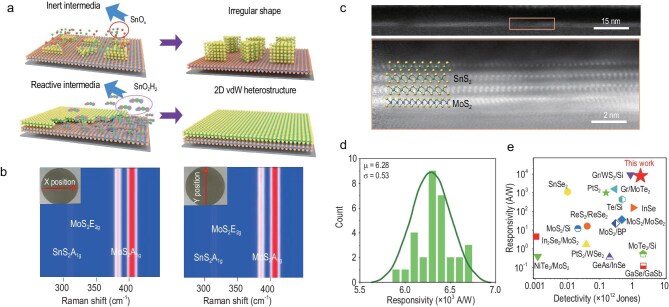
(a) Illustration of SnS_2_ growth on MoS_2_ under different reaction media: inert media result in irregular, thick SnS_2_ crystals, an impediment to film uniformity; reactive media promote the formation of SnO_2_H_2_ intermediates, enabling growth of a uniform 2D vdW heterostructure. (b) Presentation of MoS_2_ and SnS_2_ Raman spectra measured along X (left) and Y (right) positions, marking E_2__g_ and A_1__g_ modes of MoS_2_ and A_1__g_ Raman shifts (cm^−1^) of SnS_2_, a reflection of the heterostructure's vibrational traits. (c) HRTEM image display of SnS_2_/MoS_2_ atomic arrangement: the upper image (15 nm scale) shows the overall structure, and the lower magnified image (2 nm scale) reveals atomic lattice fringes, highlighting atomic-scale interfaces. (d) Histogram of 30 devices’ responsivity, with average responsivity (μ = 6.28 × 10^3^ A/W) and standard deviation (σ = 0.53), an indication of good consistency and reproducibility. (e) Comparison of responsivity and detectivity of various 2D material devices, with the red star (this work) representing this study's results, showcasing SnS_2_/MoS_2_ device superiority over others (e.g. Gr/WSe_2_, SnSe_2_). Reproduced from Ref. [6] with permission.

The resulting heterostructures exhibit pristine, defect-free interfaces, consistent crystal orientation, and exceptional uniformity—attributes vital for high-performance electronic applications. For example, Raman spectroscopy indicates that the peak shifts of MoS_2_ and SnS_2_ across the entire wafer are constrained within 0.5 cm^−1^, with intensity fluctuations limited to 2%. This demonstrates a high level of consistency in the vibrational modes of the materials throughout the wafer, suggesting homogeneous chemical structures (Fig. 1b). Moreover, the uniform thickness exceeds an impressive 99.5%, which is crucial for maintaining consistent electrical and optical properties in device applications. High-resolution transmission electron microscopy (HRTEM) further confirms the quality of these heterostructures, revealing well-defined atomic arrangements at the interfaces (Fig. 1c).

When these heterostructures are integrated into devices, they exhibit exceptional performance. They demonstrate a significant rectification effect, which is essential for applications like rectifying diodes. Furthermore, the remarkably high responsivity of 6.2 × 10³ A/W positions them as suitable candidates for optoelectronic applications, such as photodetectors (Fig. 1d). The devices also display excellent reproducibility and photo-response uniformity, which are crucial for practical applications. This consistency means that multiple devices made from these heterostructures will perform reliably, minimizing variability in device arrays and enhancing their overall performance. This study includes a comparison of the responsivity and detectivity of various 2D material devices. The results of this work are highlighted with a red star, clearly indicating that the SnS_2_/MoS_2_ device outperforms its counterparts, thus underscoring the advantages and potential of the SnS_2_/MoS_2_ device in applications based on 2D heterostructure materials (Fig. 1e).

Overall, this work represents a significant advancement in the seamless integration of 2D materials into semiconductor technologies. It successfully addresses the longstanding limitations of traditional fabrication techniques, which have often been hindered by persistent challenges such as non-uniform growth patterns and suboptimal interface quality—issues that have affected the field for years. By providing a practical, scalable, and innovative solution for growing vdW heterostructures, this approach enables the production of many emerging and desirable heterostructures at a wafer scale. This advancement is poised to enhance both fundamental research efforts and practical applications utilizing these vdW heterostructures.

## References

[1] Kwak IC, Kim J, Moon JW et al. Nat Electron 2025; 8: 235–43. 10.1038/s41928-025-01351-z

[2] Zhou Z, Hou F, Huang X et al. Nature 2023; 621: 499–505. 10.1038/s41586-023-06404-x 37674075

[3] Zhang K, She Y, Cai X et al. Nat Nanotechnol 2023; 18: 448–55. 10.1038/s41565-023-01326-1 36781997

[4] Liu L, Cai Z, Xue S et al. Nat Electron 2025; 8: 135–46. 10.1038/s41928-025-01340-2

[5] Duan X, Zhang H. Chem Rev 2024; 124: 10619–22. 10.1021/acs.chemrev.4c00586 39380397

[6] Liu S, Qin K, Yang J et al. Natl Sci Rev 2025; 12: nwaf119. 10.1093/nsr/nwaf119 40309346 PMC12042757

